# Commentary: Strong Association of the Myriad Discrete Speckled Nuclear Pattern With Anti-SS-A/Ro60 Antibodies: Consensus Experience of Four International Expert Centers

**DOI:** 10.3389/fimmu.2022.840960

**Published:** 2022-03-18

**Authors:** Werner Klotz, Manfred Herold

**Affiliations:** ^1^ Department of Internal Medicine II, Medical University of Innsbruck, Innsbruck, Austria; ^2^ Rheumatology Laboratory, Tirol-Kliniken Innsbruck, Innsbruck, Austria

**Keywords:** HEp-2 cell, ICAP, Ro/SSA, AC-4, immunofluorescence

## Introduction

International Consensus on ANA Patterns (ICAP) defines the common nuclear fine speckled pattern on HEp-2 cells assigned to code AC-4 (1). Most often, the AC-4 pattern is caused by antibodies against SS-A/Ro60. But also other antibody specificities are responsible for an AC-4 pattern. Röber and coworkers recommend an improved classification of AC-4 by describing a myriad discrete nuclear fine speckled pattern (preliminarily designated AC-4a) associated with antibodies against SS-A/Ro60 (2). Besides this pattern in sera from patients suspected of suffering from systemic autoimmune rheumatic diseases (SARD), also a plain nuclear fine speckled pattern (preliminarily designated as AC-4b) can be seen. This second subpattern of AC-4 is not related to antibodies against SS-A/Ro60 and seems to have no circumscribed autoantibody association. The authors suppose that the correct identification of the AC-4a subpattern can be useful in finding possible clinical relevance and ordering the reflex autoantibody test.

We agree with their ideas on the usefulness of recognizing specific AC-4 subpatterns with a focus on antibodies against SS-A/Ro60 and their association with connective tissue diseases. But we also think that recognizing the pattern that is not associated with antibodies against SS-A/Ro60 might be important, though up to now no specific autoantibodies or diseases are associated with the non-Ro60 subpattern.

**Figure 1:**
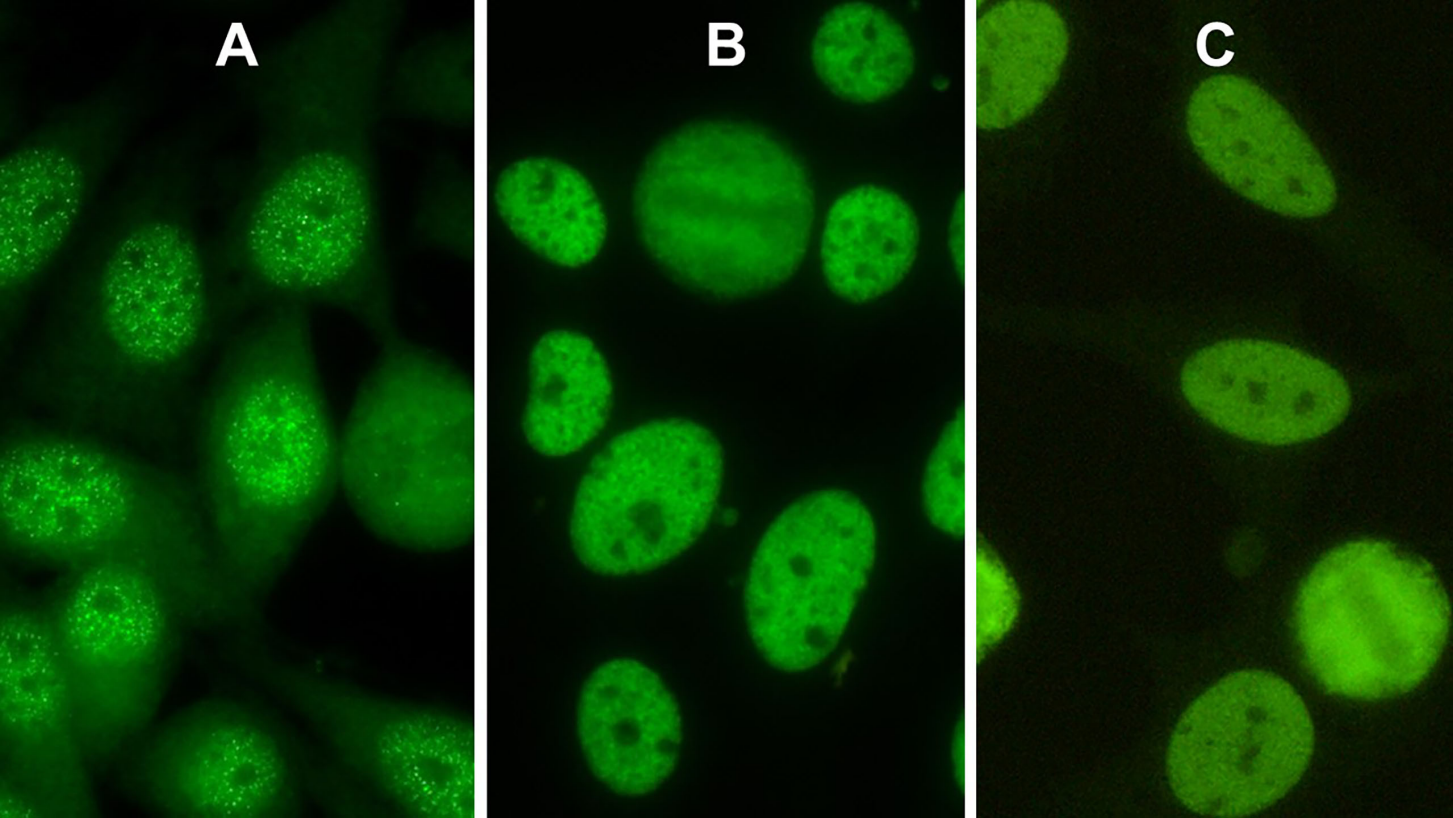
Indirect immunofluorescence pictures of HEp-2 cells. **(A)** Nuclear pattern of antibodies against Ro60 (AC-4a). **(B)** AC-4b pattern caused by antibodies against Mi-2. **(C)** AC-4 mixed pattern of antibodies against Ro60 and antibodies against Ku.

Röber et al. described the pattern caused by antibodies against SS-A/Ro60 as “a myriad discrete nuclear fine speckled pattern”. We agree with their description and confirm the picture of a nuclear fine speckled pattern characterized by a large number of small speckles different in both size and brightness. In contrast, the non-Ro60 nuclear fine speckled pattern illustrates indistinguishable fine speckles revealing an almost homogeneous appearance of the nucleoplasm (**Figure 1B**). Mixtures of both patterns may happen in sera of patients containing more than one autoantibody specificity (**Figure 1C**).

We agree that the non-Ro60 plain fine speckled AC-4 nuclear pattern ought to be described and characterized specifically to differ it from the AC-4 Ro60 pattern and to direct our thoughts to antibodies not found by usual antinuclear antibody (ANA) screening tests.

## Clinical Importance of AC-4 Subgroups

The AC-4 nuclear fine speckled pattern may be present in different SARD (3). Recognizing the specific AC-4 subpatterns might be helpful to find the clinical diagnosis and choose the best reflex autoantibody test to confirm the autoantibody subtype. Autoantibodies to SS-A/Ro are part of the current classification criteria for Sjogren’s syndrome (SjS), though the criteria do not distinguish between Ro60 and Ro52/TRIM21 antibodies (4). The AC-4 SSA/Ro60 pattern can be identified with high probability by experienced observers and may be confirmed by specific immunoassays or a common extractable nuclear antigen (ENA) test, which includes SS-A 60-kD antigen. Since Röber’s report, we distinguish both subpatterns in our laboratory with high probability, but we do not yet mention the result on the patient’s report. If the AC-4 pattern is not caused by antibodies against SS-A/Ro60, no specific autoantibodies can be found using common ENA tests except antibodies against SS-B/La, which reveal an AC-4 plain fine speckled pattern preliminarily defined as AC-4b by Röber et al. Isolated antibodies against SS-B/La are very rare and have no diagnostic relevance (5).

Physicians asking for ANA and subtypes usually are not aware that routine testing on ANA subtypes only includes a restricted number of possible autoantibodies but does not include autoantibodies associated with inflammatory myositis like Mi-2, TIF1γ, Ku, or rare antibody specificities seen in other connective tissue diseases. Antibodies against RNA polymerase III also reveal an AC-4 pattern different from the AC-4 SSA/Ro60 pattern. Anti-RNA polymerase III antibodies are a specific marker for systemic sclerosis, associated with severe disease with major organ and diffuse cutaneous involvement (6, 7) and seems to be strongly correlated with concomitant scleroderma and cancer (8).

Physicians might be misdirected if they receive a positive ANA test on HEp-2 cells with an AC-4 pattern but a negative ENA screening. If the laboratory doing the ANA test is informed about suspicious clinical diagnoses, follow-up tests other than ENA tests might be useful. Assays for myositis or scleroderma-related antibodies are indicated in patients showing an AC-4 non-Ro60 pattern.

## Discussion

The nuclear fine speckled pattern on HEp-2 cells with ICAP’s code AC-4 can be seen frequently in daily routine workup of autoantibody diagnostics. It is well known that AC-4 and the description nuclear fine speckled include different subpatterns that might be recognized and distinguished by experienced assessors. On ICAP’s webpage, some more help to differ the two subpatterns is included as a note to pattern AC-4 with additional pictures (https://www.anapatterns.org/view_pattern.php?pattern=4).

One of these subpatterns with small distinct dot-like speckles different in size and brightness is caused by antibodies against SS-A/Ro60 (**Figure 1A**). In contrast, the second AC-4 subpattern presents a uniform distribution of equal-sized fine speckles in the nucleoplasm (**Figure 1B**) and is not associated with antibodies against SS-A/Ro60. **Figure 1B** shows a patient’s serum with monospecific Mi-2 antibodies.

Röber et al. (2) preliminarily designated these patterns as AC-4a and AC-4b. It might be considered to subclassify AC-4 into AC-4.1 and AC-4.2 to keep the numerical order of the decision tree. But we agree with Röber et al. that AC-4 subpatterns should be described in detail to facilitate the decision on the further workup of AC-4 positive samples, on additional reflex tests, and better help in the diagnostic workup of difficult clinical cases.

## Author Contributions

WK together with MH had the idea for this commentary. Together, they wrote the manuscript and collected the pictures. All authors listed have made a substantial, direct, and intellectual contribution to the work and approved it for publication.

## Conflict of Interest

The authors declare that the research was conducted in the absence of any commercial or financial relationships that could be construed as a potential conflict of interest.

## Publisher’s Note

All claims expressed in this article are solely those of the authors and do not necessarily represent those of their affiliated organizations, or those of the publisher, the editors and the reviewers. Any product that may be evaluated in this article, or claim that may be made by its manufacturer, is not guaranteed or endorsed by the publisher.

## References

[1] Chan EK Damoiseaux J Carballo OG Conrad K de Melo Cruvinel W Francescantonio PL. Report of the First International Consensus on Standardized Nomenclature of Antinuclear Antibody HEp-2 Cell Patterns 2014-2015. Front Immunol (2015) 6:412. doi: 10.3389/fimmu.2015.00412 26347739 PMC4542633

[2] Röber N Dellavance A Ingénito F Reimer M-L Carballo OG Conrad K. Strong Association of the Myriad Discrete Speckled Nuclear Pattern With Anti-SS-A/Ro60 Antibodies: Consensus Experience of Four International Expert Centers. Front Immunol (2021) 12:730102. doi: 10.3389/fimmu.2021.730102 34675922 PMC8524051

[3] Damoiseaux J Andrade LEC Carballo OG Conrad K Francescantonio PLF Fritzler MJ. Clinical Relevance of HEp-2 Indirect Immunofluorescent Patterns: The International Consensus on ANA Patterns (ICAP) Perspective. Ann Rheum Dis (2019) 78:879–89. doi: 10.1136/annrheumdis-2018-214436 PMC6585284 30862649

[4] Shiboski CH Shiboski SC Seror R Criswell LA Labetoulle M Lietman TM. American College of Rheumatology/European League Against Rheumatism Classification Criteria for Primary Sjögren’s Syndrome: A Consensus and Data-Driven Methodology Involving Three International Patient Cohorts. Ann Rheum Dis (2017) 76(1):9–16. doi: 10.1136/annrheumdis-2016-210571 27789466

[5] Baer AN McAdams DeMarco M Shiboski SC Lam MY Challacombe S Daniels TE. The SSB-Positive/SSA-Negative Antibody Profile Is Not Associated With Key Phenotypic Features of Sjögren’s Syndrome. Ann Rheum Dis (2015) 74(8):1557–61. doi: 10.1136/annrheumdis-2014-206683 PMC6697481 25735642

[6] Conrad K Schössler W Hiepe F Fritzler MJ. Autoantibodies in Systemic Autoimmune Diseases: A Diagnostic Reference, Vol 2, 3rd Edition. Lengerich: Pabst Science Publishers (2015). 359 p.

[7] Cavazzana I Ceribelli A Airo P Zingarelli S Tincani T Franceschini F. Anti-RNA Polymerase III Antibodies: A Marker of Systemic Sclerosis With Rapid Onset and Skin Thickening Progression. Autoimmun Rev (2009) 8(7):580–4. doi: 10.1016/j.autrev.2009.02.002 19393210

[8] Moinzadeh P Fonseca C Hellmich M Shah AA Chighizola C Denton CP. Association of Anti-RNA Polymerase III Autoantibodies and Cancer in Scleroderma. Arthritis Res Ther (2014) 16(1):R53. doi: 10.1186/ar4486 24524733 PMC3978927

